# Clinical and Patient-Reported Functional Outcome of Semitendinosus Autograft Anterior Cruciate Ligament Reconstruction With FiberTape® InternalBrace™ All-Inside Technique: A Prospective Study

**DOI:** 10.7759/cureus.44700

**Published:** 2023-09-05

**Authors:** Manu Bora, Prithviraj Deshmukh

**Affiliations:** 1 Orthopedic Surgery, Nexus Day Surgery Center, Mumbai, IND

**Keywords:** acl reconstruction, lachman test, vas score, womac score, koos score

## Abstract

Aim: The purpose of this study is to report the early one-year clinical and patient-reported functional outcomes of semitendinosus autograft anterior cruciate ligament reconstruction with the FiberTape® InternalBrace™ all-inside technique.

Materials and methods: The patient-reported functional outcomes and clinical outcomes were analyzed prospectively following the treatment of 324 unilateral ACL rupture cases with the FiberTape® InternalBrace™ Technique. Patient-reported outcome measures (KOOS scores, WOMAC scores, VAS score, and IKDC score) and clinical examinations like pre-operative and post-operative pivot shift, Lachman test, and range of motion (ROM) were conducted. These tests and examinations were recorded for each patient before surgery and at 3, 6, and 12 months after surgery. Secondary outcomes like re-rupture, infection, synovitis, or limited range of motion were noted. The mean follow-up period was 18±4.5 months (range: 12-24 months).

Results: Out of a total of 324 cases, 37 cases (11.4%) could not be followed up. 158 patients (55.1%) were male and 129 were female (44.9%). Preoperative mean KOOS pain value, KOOS symptoms, KOOS ADLs, KOOS sport and recreation, and KOOS quality of life were 77.05, 78.69, 84, 21, 89.7, and 57, respectively. All KOOS subsections increased significantly at one-year to 98.37, 99.09, 98.95, 99.02, and 99.30 (p<0.0001), respectively. Mean preoperative WOMAC pain, WOMAC stiffness, and WOMAC function were 76.4, 65.2, and 74.1 and increased significantly at one-year to 94.5, 89.6, and 98.2 (p<0.0001), respectively. There was a significant decrease in VAS for pain from 2.93 before surgery to 0.12 (p<0.0001) at one year. The IKDC score significantly changed from a pre-treatment value of 50.9 to 96.2 (94.5-97.8) at a follow-up of one year. The Lysholm score at 12-months was significant at 95 (93.1-96.9), p<0.05. Post-operative Lachman test values decreased significantly, which meant decreased laxity, from 1.98 (1.89-2.07) pre-operative to 1.60 (1.57-1.62) p<0.05 at one-year post-operative. At one-year follow-up, 276 patients (96%) had fully recovered.

Conclusion: It was concluded that the FiberTape® InternalBrace™ technique for ACL reconstruction provides orthopedic surgeons with an effective alternative technique to conventional methods of surgery and also reduces the overall time to recovery for patients, thereby allowing them to return to sports faster.

Level of evidence: Level 4

## Introduction

Anterior cruciate ligament injury is one of the most frequently observed knee injuries, with an incidence of more than 200,000 cases globally and reconstruction of 100,000 cases annually. It was observed that 70% of ACL injuries occur because of non-contact injuries, while 30% of cases were due to direct contact [1,2]. Conventional ACL surgery has a revision rate ranging from 1.7% to 7.7%, with 35% of such cases having isolated trauma causing first-time graft failures [3]. Biological failures may occur during the period while the graft is highly sensitive, even though there may be no traumatic accidents or technical errors due to inadequate graft "ligamentization" [4]. Histological stages for the graft involve necrosis, revascularization, cellular repopulation, proliferation, and collagen remodeling. The last collagen remodeling stage comprises a change in the non-reducible and reducible cross-link ratio in collagen fibrils and goes on throughout the first year after surgery [5,6]. Hence, it is crucial to protect the graft during these phases when mechanical loads are applied within the limit and in a controlled manner over the graft so that the possibility of mechanical and biological failure is reduced. Therefore, a protective technique like internal suture augmentation could become a "safety belt" for grafts and prove their effectiveness [7].

The all-inside technique involving the use of a dual retro-cutter (Arthrex, Naples, FL) was described by Lubowitz in 2006. This allowed drilling in anatomical antegrade as well as retrograde femoral and tibial tunnels following its intra-articular assembly. Lately, inside-out tunnel drilling has been made easy by the use of FlipCutter® (Arthrex, Naples, FL) [8,9].

InternalBrace™ technique (IBLA, Arthrex, GmbH, Naples, FL) is a technique involving ligament reconstruction augmentation using suture tape, which supports or braces the ligament, thereby acting as a secondary stabilizer. The ligament, in turn, stays protected, and in the healing phase, this also promotes natural healing, which allows early mobilization [10]. FibreTape® (Arthrex, Naples, FL) is a suture tape made of braided ultra-high-molecular-weight polyethylene material. In the graft healing phases, this provides biomechanical support by acting as reinforcement [11]. This suture tape augmentation was used successfully with Brostrom repair and postero-medial corner repair [12,13]. Samuel Bachmaier conducted a study that tested the use of internal suture augmentation with a biomechanical full-construct hamstring tendons model. It was concluded that there is an increase in ultimate load to failure as well as dynamic stiffness with suture tape augmentation, and the augmented sample has decreased dynamic elongation [14].

The objective of this study was to report the clinical results of the pre- and post-operative Lachman test, pivot shift test, range of motion (ROM), and patient-reported functional outcomes (KOOS score, WOMAC score, VAS score, and IKDC score) among patients following semitendinosus autograft ACL reconstruction using the FiberTape® InternalBrace™ technique. We hypothesized that the patient-reported functional outcome values would significantly improve at one year.

## Materials and methods

Study design

This prospective cohort study was conducted in multiple hospitals and centers by a senior surgeon (Dr. Manu Bora). Pre-operative and post-operative examinations, functional assessments, and evaluations were performed by a senior resident doctor (Dr. Prithviraj Deshmukh).

Patients were prospectively evaluated using the Surgical Outcome System (SOS, Arthrex, Naples, FL, USA). Informed consent was obtained from the patient preoperatively for the study. Questionnaires, e-mails, and patient-reported functional outcome measures (PROMs) were sent through SOS, which is a web-based tool, at pre-planned time intervals. Permission to collect patient data was obtained from the medical ethics board (IECNDSC/SS03/2019-20).

Study population

From September 2019 until July 2021, 324 patients with ACL tears fulfilled the inclusion criteria, and all-inside ACL reconstruction with the FiberTape® (Arthrex) InternalBrace™ technique was performed on them.

Inclusion Criteria

Patients in the age range of 13-60 years were included. Patients were diagnosed with an ACL tear if they had previous knee trauma. If any other associated injury was present along with the ACL involvement, only the following clinical situations were present: partial resection of a meniscus tear, a small but stable tear in the meniscus treated with fixation, cartilage lesions diagnosed using MRI with no additional pathologic intraoperative findings, and an ACL tear detected radiologically (MRI). Cases included had clinical evaluations done, like the pivot shift test and the Lachman test, to confirm. 

Exclusion Criteria

Cases that did not meet the inclusion criteria were cases of previous ACL surgery on the concerned knee, prominent articular surface injury, ACL injuries on both knees, neuromuscular disorders, and malalignment in patients (Genu valgum, Genu varum, and Genu recurvatum). Cases were also excluded if they had the presence of extra-articular or intra-articular knee injuries like injury to the posterior cruciate ligament (PCL), complete medial collateral ligament (MCL), or lateral collateral ligament (LCL) as detected in an MRI or an unstable longitudinal meniscus tear repair in which further post-operative treatment could interfere with the standard rehabilitation protocol.

Study outcomes

Primary outcomes for the study: KOOS score, WOMAC score, VAS score, and IKDC score, along with clinical examinations like preoperative and postoperative pivot shift tests, preoperative and postoperative Lachman tests, and range of motion (ROM). These assessments were made for each patient preoperatively and 3, 6, and 12 months postoperatively. The secondary outcomes recorded were cases of re-rupture, synovitis, infection, or restricted ROM. 

Study intervention

Room setup and equipment setup were done, and the patient was placed in a supine position. The placement of the thigh tourniquet was done, and the leg of the patient was positioned in such a way that it was close to the side edge of the table so that while operating, there was no need for leg support, and the leg would hang down in flexion from the side of the table. While the opposite leg stayed in extension.

The operative leg was kept such that it could flex to at least 120 degrees. After scrubbing, painting, and draping, diagnostic arthroscopy was done. After approaching the joint through anterolateral and anteromedial portals, all three compartments were visualized (patello-femoral joint, medial compartment, and lateral compartment), and the anatomic footprint of the torn ACL was visualized. The ACL remnant was then shaved off from the intercondylar notch and tibial spine with the help of an Excalibur shaver blade at a frequency of 3000 rpm for identification of the ACL footprint on the tibia and femur. The femoral footprint was marked by partially drilling a single cortex with the help of a guide pin and keeping the knee flexed at 120 degrees. This position is usually marked 6-7 mm ahead of the back wall.

Graft Harvesting

Before giving the incision, the pes anserine was palpated, which is usually found with three fingers distal to the joint line and two fingers medial to the tibial tubercle, following which a 3 cm incision was made. Soft tissue dissection was done until the sartorial fascia; below it, the pes anserine was palpated. Usually, a blunt instrument or Metzenbaum scissors were slid below the sartorial fascia to elevate the fascia from the superior border. The advantage of this technique is that it protects the MCL from any trauma as it lies deep in the fascia. After the elevation of the fascia, a longitudinal incision was made to expose the gracillis and semitendinosus tendons. The semitendinosus was isolated and whipstitched with a 3.0 ethibond suture. Releasing of all the adhesions was done, and a good recoil was seen on the application of tension. This confirmed that all the adhesions had been cleared, following which an atraumatic tendon stripper was used to harvest the tendon by applying an opposite pull against the stripper. The knee was kept in flexion to avoid a saphenous nerve injury.

Paper Graft Preparation

The semitendinosus was used to create the GraftLink construct. After assembling the attachments on the GraftPro® graft preparation set, the ACL TightRope® II internal brace implant was placed over the attachments of the GraftPro ® set. The distance between the TightRope® II internal brace loop was measured to be equal to or 10 mm less than the graft length.

Note: Usually, a 6.5 cm quadrupled Graft link will require 26 cm of total length of graft for its preparation, out of which 2 cm will be inside the femur, and 2 cm will be in the tibia.

The graft was then placed and arranged through the implants by layering it symmetrically around the loops. The ends of the graft were stitched with 1.3-mm Fiberwires®, and Fibertape® was loaded around the graft after passing it through the ACL TightRope II internal brace implant. The stitch’s one end was passed over and under the graft loop to ensure the graft was tucked inside the loop; further tensioning tapered the end of the graft and gave it a uniform cylindrical shape with tapering ends.

Drilling the Femoral Tunnel

By flexing the knee at 12 degrees over the previously marked area, the guide pin was again placed after attaching it to the transportal ACL formal guide through the medial portal on the medial aspect of the lateral femoral condyle (previously marked). After drilling with the help of an AR 400 drill, the intraosseous length was marked, and a flexible reamer was used for reaming. A 3.0 ethibond suture was passed through the drill hole, and if required, even FlipCutter III was also used to make the femoral socket.

Drilling the Tibial Tunnel

No separate incision was made for tibial tunnel drilling. A tibial guide was placed through the previously made graft harvest incision and anteromedial portal. The jig was placed on the tibial ACL footprint along with the medial tibial spine, which somewhat corresponded to the anterior horn of the lateral meniscus. The sleeve is tightly attached to the bone, and then drilling is done forward with the help of FlipCutter III up into the joint, following which a rubber marker was used as a marker, and then the end of the flip cutter was flipped. Back-word drilling was done once the drilling was completed up to the desired length. The flip cutter was manually pushed forward into the joint, and the tip was unflipped and removed from the joint, following which, in a similar fashion to the femur, even from the tibial hole, a 3.0 ethibond suture was passed. Both the sutures from the femur and tibia were retrieved from the anteromedial portal, and TightRope® III internal brace loop shortening strands were passed through the suture on the femur side. The suture was pulled through the opposite side, and shortening strands were pulled until the button was completely passed out through the femoral tunnel. The fixation of the button was confirmed by pulling the graft in the opposite direction. Next, the graft was pulled into the femoral sockets. Shortening strands of TightRope® III internal brace was pulled one by one up to the markings made on the femoral end. Similarly, the step was repeated for the tibial end, and the TightRope® III internal brace was pulled out through the tibial tunnel.

Loading of ABS Button; SwiveLock

Once the TightRope II and the graft were passed through the femoral tunnel, the placement of the graft was confirmed by pulling the TightRope II distal strands back firmly. This confirmed that the loop of TightRope II has been flipped and is getting obstructed over the entry point of the femoral tunnel. The suture, which was previously retrieved through the tibial tunnel, was loaded with the distal strands of TightRope II, which had a preassembled ABS implant passing suture, and the FiberTape®️. The retrieving sutures that were passed through the tibial tunnel were then pulled distally through the tibial tunnel to advance both the TightRope®️ II out of the tibia distally. Then, the graft is advanced into the tunnel by pulling the shortening strands of TightRope II. Once the graft was passed transtibial into the socket made by a flip cutter, the 14-mm concave ABS button implant was loaded onto the loop by passing each side through its respective slot on the button through the shortening strands of the TightRope®️ II internal brace and FiberTape®️ sutures. The shortening strands were then pulled one by one, and the button was advanced toward the bone. While pulling the white shortening strands to advance the button to the bone, the position of the knee was kept in extension. In the end, just when the button was about to touch the periosteum, it was ensured that the button had a clear path to the bone and no soft tissue was entrapped under the button.

After the soft tissue above the bone was cleared out, a hole was drilled into the tibia with the help of a spade tip drill bit, which was approximately 25 mm in depth, and the hole was tapped with a 5.2 mm tap. While performing this, the knee was kept in full extension. Maintaining the same position of the knee, the FiberTape®️ suture and graft and whipstitch sutures were passed through the eyelet of the 4.75 mm PEEK SwiveLock®️ anchor. An oversized tap of 5.2 mm for a 4.75 mm anchor was used to average the hard bone density and also to accommodate 2 strands of FiberTape®️ suture; hence, a 5.2 mm tap was recommended for harder bone or when more than two tapes are used. Once the FiberTape suture tails were loaded through the eyelet of the 4.75 mm PEEK SwiveLock®️ anchor, The anchor was pushed into the drill hole until the eyelet was fully seated into the bone while maintaining the tension on the suture limbs of fiberglass tape and hammering the PEEK anchor into the tibia. Tightening of the PEEK anchor along with strands of FiberTape into the tibia was done. 

Once this procedure was completed, the graft was fully tensioned by pulling the shortening strands of TightRope II, and a posterior drawer force of 50-60 N was applied to the newly made construct for 10 minutes with 30 degrees of knee flexion. The knee was cycled 25 times once the tension was given adequately, and both sides of the TightRope II shortening strands were pulled one by one to confirm the proper tensioning of the graft link construct. TightRope II distal shortening strands are tied over the button to close the TightRope loop.

Wound Closure

After giving a thorough wash with saline intra- and extra-articular, the harvest site was closed in layers (sartorial fascia, subcutaneous tissue) with 3.0 cutting edge vicryl sutures and skin and the portal site with 3.0 cutting edge ethillion. A compression dressing was applied to avoid post-operative swelling.

Postoperative Protocol

Postoperatively, the patient was allowed a complete range of motion and full weight-bearing based on his tolerance until he was able to demonstrate proper leg control and well-functioning quadriceps muscles. While recommending an exercise rehabilitation program and providing management guidance for each phase, clinicians should adopt a clinical reasoning approach. For each step of the ACL protocol, programs were adjusted according to the individual case scenarios.

The ACL rehab protocol is divided into five phases. In phase one, which was immediately after surgery, patients were made to do a range of motion exercises like low lead extension and long duration stretching (5 min), like heel prop, prone hang, calf stretch, and hamstring stretch and flexion up to the 90° limit. The patient was also made to do strengthening exercises for quadriceps, hamstrings, hip abductors, adductor strengthening, and ankle pumps with TheraBand®. Phase two, which was known as early rehabilitation, was started from 3-6 weeks.

In this phase, along with the muscle strength, the goal was to progress in neuromuscular retraining, and hence resistance exercises were also added to the above-mentioned exercises of phase one. Phase three, which was known as the Strengthening and Control phase, was started at 7-16 weeks. In this phase, the patient was made to run and hop. If the patient had no pain, swelling, or instability, then exercises progressed to the next phase. Usually, after phase three, individuals got back to their regular routine, and the remaining two phases were only for professional athletes who wanted to return to their sports. In phase four, advanced training was started after 16-20 weeks of operation, in which patients were made to do agility drills at 50% to 75% of their efforts and speed. In phase six, which lasted from 20 to 2 weeks or more, athletes were made to do sports-specific drills. The patient was able to get back to full activity within six months postoperatively.

Follow-Up

Follow-up with the patient was done at two weeks, three, six, and 12 months. The results of the KOOS score, WOMAC score, VAS score, and IKDC score, along with clinical examinations like the Lachman test and range of motion (ROM), were recorded at three, six, and 12 months. Any case with re-rupture, synovitis, infection, or restricted ROM was noted, and appropriate interventions were done for them.

Statistical analysis

The data was evaluated in Microsoft Office 2007 and analyzed using IBM Corp. Released 2017. IBM SPSS Statistics for Windows, Version 25.0. Armonk, NY: IBM Corp. The demographics and clinical outcomes were summarized using descriptive statistics. The means value +/- standard deviations with ranges was recorded for each category. A comparison of the preoperative and postoperative patient-reported functional outcome values was done, followed by confirmation of the normal distribution of the data using the Shapiro-Wilk test. All pairs were confirmed using Tukey-Kramer testing. Results were inferred to be significant if the p-value was less than 0.001 (p<0.001).

## Results

Between September 2019 and July 2021, 324 patients suffering from acute proximal ACL tear underwent all-inside semitendinosus autograft ACL reconstruction with the FiberTape® (Arthrex) InternalBrace™ Technique. Thirty-seven cases were lost to follow-up (11.4%), making the total number of patients 287. One hundred and fifty-eight patients (55.1%) were male and 129 were female (44.9%). The mean age of patients included in the study was 36.5±14.8 years (range: 13-60). The mean follow-up period in our study was 18±4.5 months (range: 12-24 months). Demographic data and initial clinical assessment characteristics were recorded, as given in Table 1.

**Table 1:** Demographics of the patients included in the study and initial manual assessment.

Demographics	Value
Total no. of patients	287
Age	36.5 ± 14.8 years (range: 13-60)
Right side to left side injury distribution (%)	202 patients (70.4%): 85 patients (29.6%)
Time since injury (months)	8.65 ± 8.99 (range: 7-34)
Male to female distribution (%)	158 patients (55.2%): 129 patients (44.9%)

Knee injury and osteoarthritis outcome score (KOOS)

**Figure 1:**
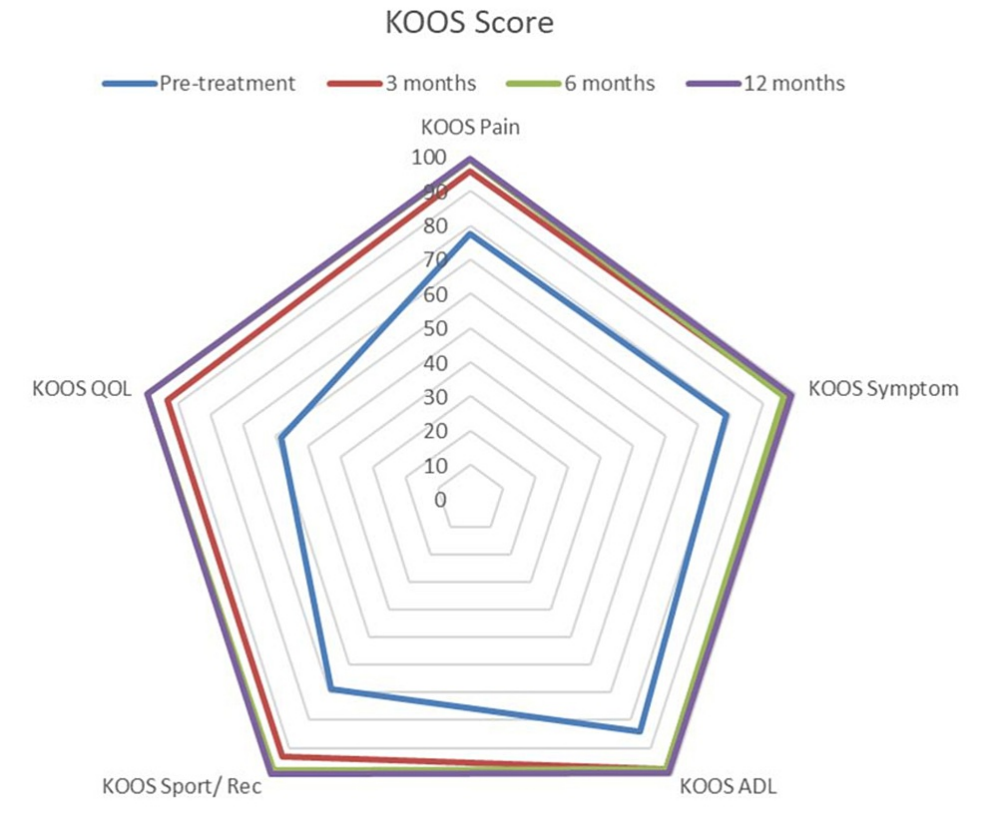
Spider chart depicting significant improvements at different follow-up times in the KOOS score involving different sub-sections. KOOS: Knee Injury and Osteoarthritis Outcome Score.

At one-year follow-up, KOOS for pain, symptoms, ADLs, sports and recreation, and quality of life all showed significant improvements (p<0.0001) (Figure 1).

**Figure 2:**
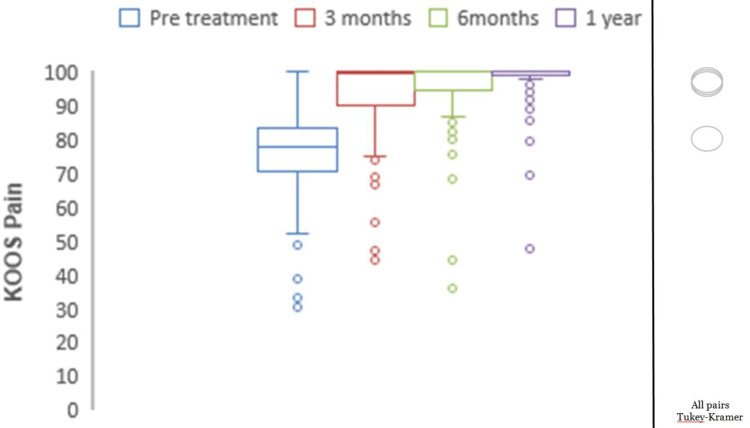
Chart depicting the increase in KOOS for pain with time. KOOS: Knee Injury and Osteoarthritis Outcome Score. *all pairs Tukey-Kramer testing p-value < 0.05 (statistically significant).

**Figure 3:**
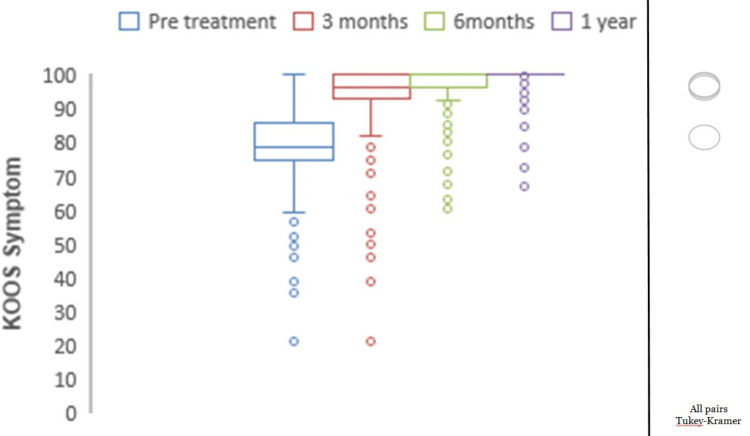
Chart depicting the increase in KOOS for symptoms with time. KOOS: Knee Injury and Osteoarthritis Outcome Score. *all pairs Tukey-Kramer testing p-value < 0.05 (statistically significant).

**Figure 4:**
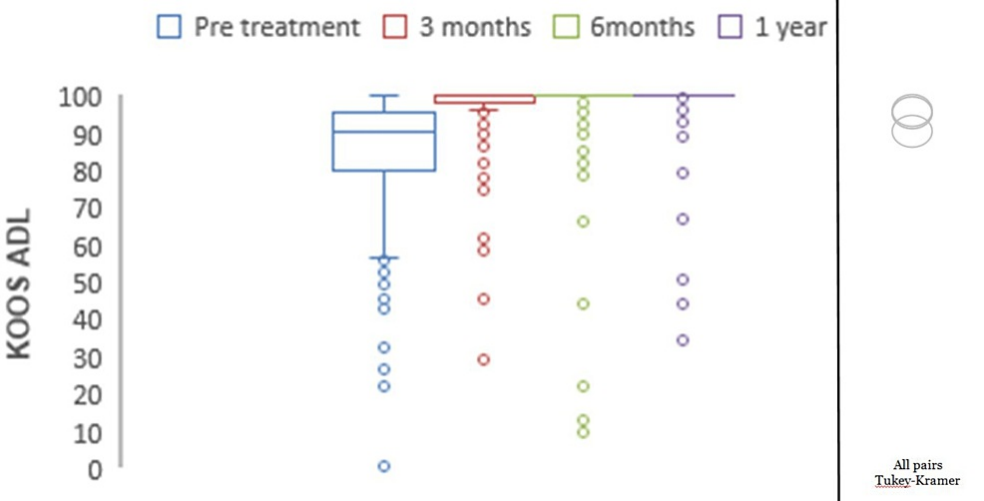
Chart depicting the increase in KOOS for ADLs with time. KOOS: Knee Injury and Osteoarthritis Outcome Score. *all pairs Tukey-Kramer testing p-value < 0.05 (statistically significant).

**Figure 5:**
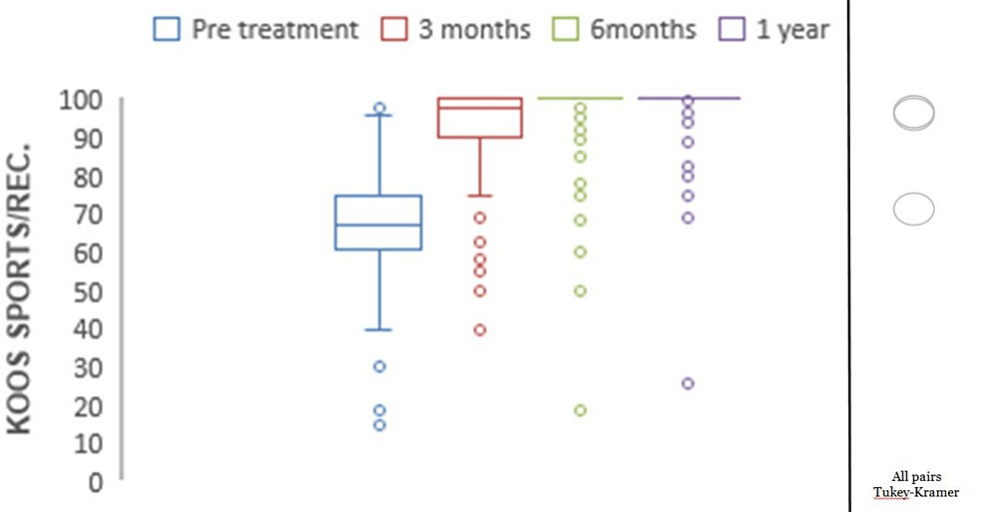
Chart depicting the increase in KOOS for sports and recreation with time. KOOS: Knee Injury and Osteoarthritis Outcome Score. *all pairs Tukey-Kramer testing p-value < 0.05 (statistically significant).

**Figure 6:**
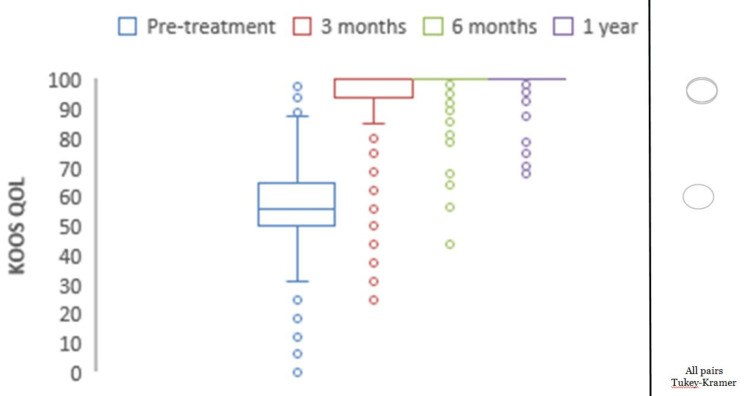
Chart depicting the increase in KOOS for quality of life with time. KOOS: Knee Injury and Osteoarthritis Outcome Score. *all pairs Tukey-Kramer testing p-value < 0.05 (statistically significant).

Mean preoperative KOOS pain was 77.05, which showed significant improvements with mean scores of 94.02 at three months, 96.45 at six months, and 98.37 at one year (p<0.0001) (Figure 2). The mean preoperative KOOS symptoms were 78.69, which showed significant improvements with mean scores of 95.16 at three months, 96.51 at six months, and 99.09 at one year (p<0.0001) (Figure 3). The mean preoperative KOOS ADLs were 84.21, which showed significant improvements with mean scores of 97.73 at three months, 97.79 at six months, and 98.95 at one year (p<0.0001) (Figure 4). The mean preoperative KOOS Sports and Recreation score was 89.7, which showed significant improvements with mean scores of 93.25 at three months, 97.92 at six months, and 99.02 at one year (p<0.0001) (Figure 5). The mean preoperative KOOS Quality of Life was 57, which showed significant improvements with mean scores of 93.12 at three months, 98.01 at six months, and 99.30 at one year (p<0.0001) (Figure 6). Significant differences involving any of the KOOS subsections between three-month, six-month, and one-year follow-ups were not observed. 

Western Ontario and McMaster universities osteoarthritis index (WOMAC)

**Figure 7:**
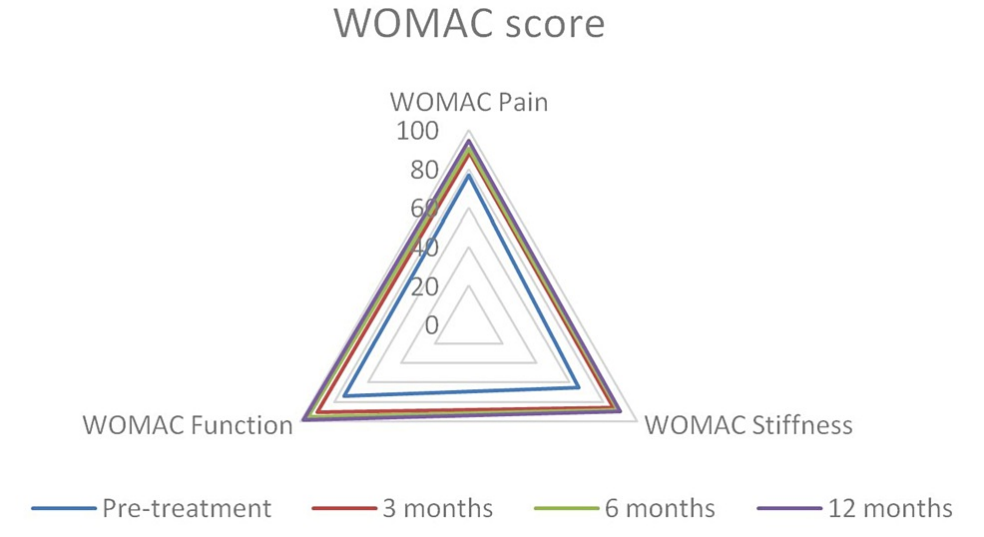
A spider chart depicts significant improvements at different follow-up times in WOMAC for pain, function, and stiffness. WOMAC: Western Ontario and McMaster Universities Osteoarthritis Index.

At one-year follow-up, WOMAC for pain, function, and stiffness all showed significant improvements (p<0.0001) (Figure 7).

**Figure 8:**
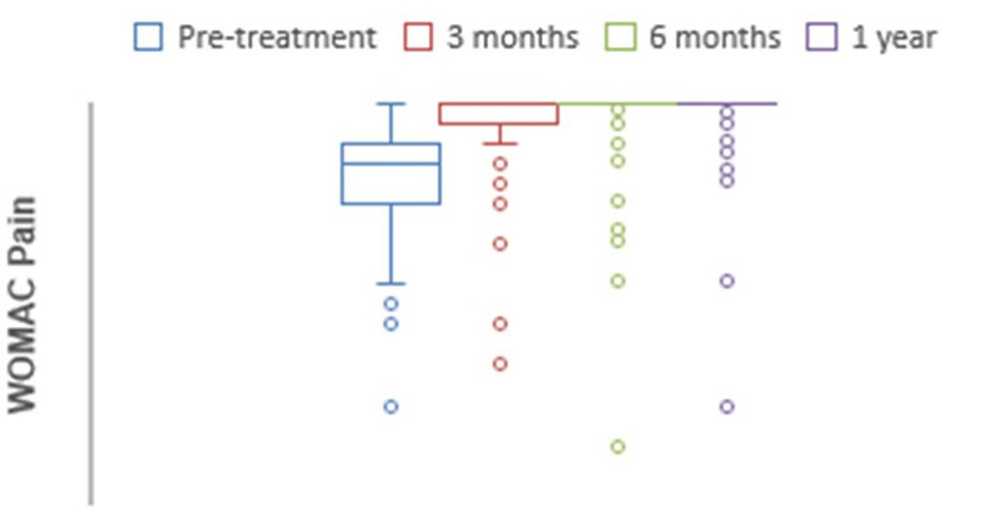
Chart depicting the increase in WOMAC pain scores with time. WOMAC: Western Ontario and McMaster Universities Osteoarthritis Index.

**Figure 9:**
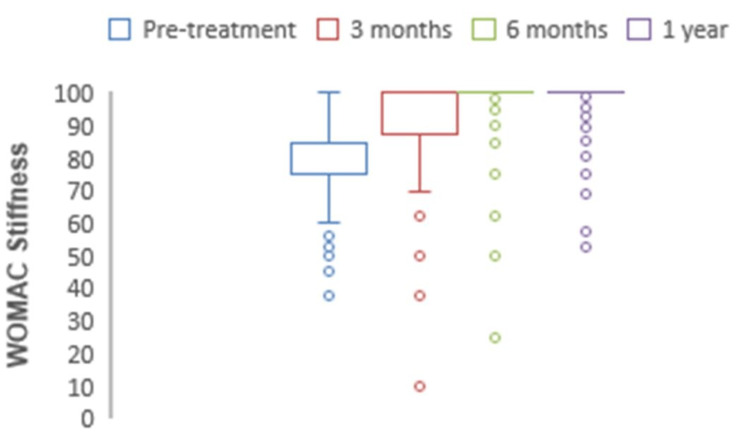
Chart depicting the increase in WOMAC stiffness scores with time. WOMAC: Western Ontario and McMaster Universities Osteoarthritis Index.

**Figure 10:**
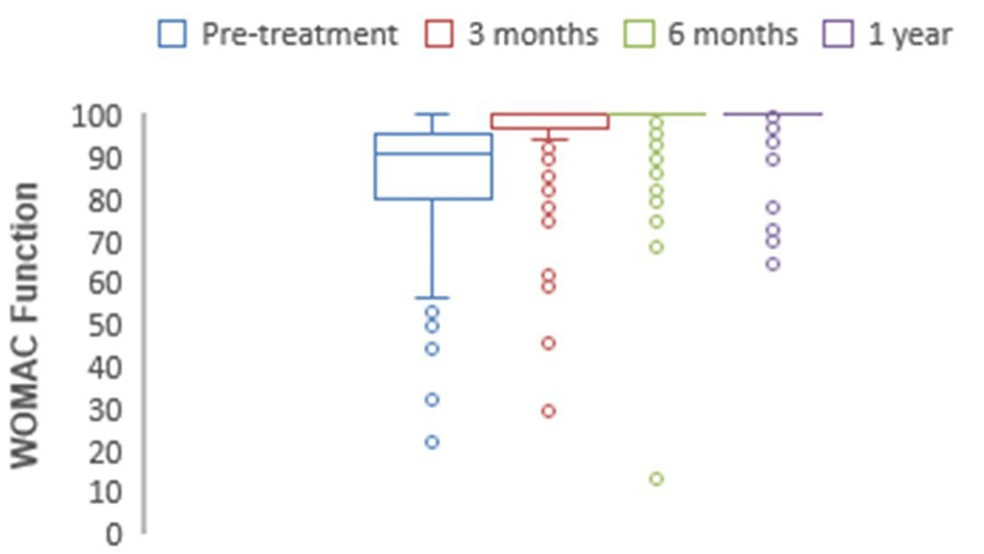
Chart depicting the increase in WOMAC function scores with time. WOMAC: Western Ontario and McMaster Universities Osteoarthritis Index.

The mean preoperative WOMAC for pain was 76.4, which at one-year follow-up significantly increased to 94.5 (p<0.0001) (Figure 8). The mean preoperative WOMAC for stiffness was 65.2, which at one-year follow-up significantly increased to 89.6 (p<0.0001) (Figure 9). The mean preoperative WOMAC for function was 74.1, which at one-year follow-up significantly increased to 98.2 (p<0.0001) (Figure 10). The WOMAC subsections at different postoperative time intervals showed no significant differences.

Visual analog pain scale (VAS)

**Figure 11:**
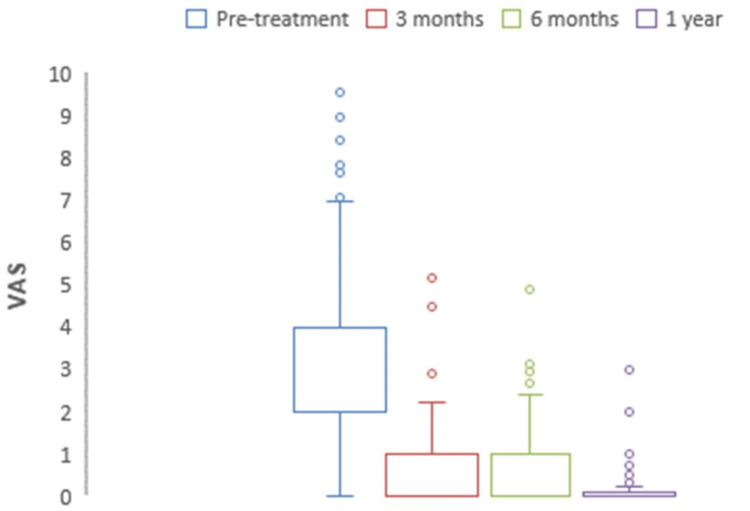
The chart depicting a decrease in preoperative to one-year postoperative VAS pain scores is significant. VAS: Visual Analog Scale.

The mean preoperative VAS for pain scores decreased significantly from 2.93 to 0.12 at one-year (p<0.0001) (Figure 11). At three months, six months, and one-year postoperative time intervals, no significant differences were observed.

International knee documentation committee (IKDC)

**Figure 12:**
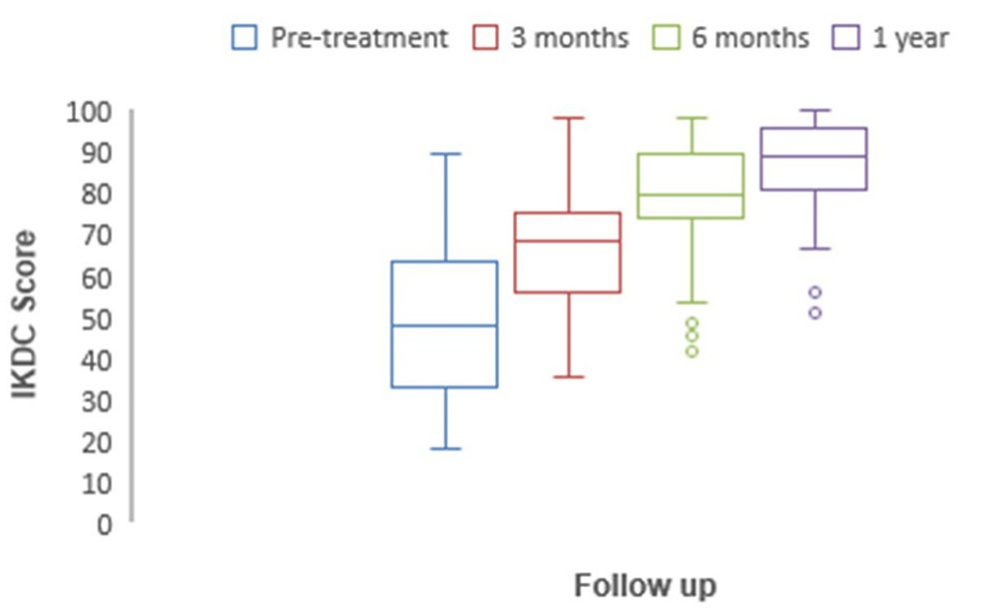
The chart depicting the increase in preoperative to one-year postoperative IKDC scores is significant. IKDC: International Knee Documentation Committee.

At one-year follow-up, the IKDC score significantly changed from a pre-treatment value of 50.9 to 91.2 (p < 0.05). It was 50.9 (47.8-55) pre-operatively, 69.8 (68.3-71.2) at three months, 82.7 (79.8-85.6) at six months, and 96.2 (94.5-97.8) at follow-up of one year (Figure 12).

Patient reported outcomes

The pre-pivot test and pre-Lachman test with grading were recorded. Most of the patients were in grade 2 (57%). Patients with high-grade laxity were not included, which meant chronic ACL injuries associated with meniscus tears or other associated injuries that would lead to a poor prognosis were not included in the study. A pre-operative Lysholm score of 51.0 (48.1-53.8), 74.6 (71.6-77.6) at three months, 81.2 (79.7.5-82.6) at six months, and 95 (93.1-96.9) at one year was recorded. The Lysholm score at one year was significant as compared to pre-operative (p<0.05). The preoperative Lachmeter score was 1.98 (1.87-2.08). The Lachman test postoperatively at three, six, and 12 months showed a significant decrease in laxity, with the Lachman test values being 1.93 (1.82-2.05), 1.82 (1.70-1.94), and 1.60 (1.57-1.62), respectively (p<0.05). Range of motion (ROM) was recorded preoperatively and at three, six, and 12 months postoperatively. ROM ranging from 0 (full extension) to 135 (knee flexion) was considered normal. A normal range of motion was achieved at one year, with the mean ROM being 132.3 (130.2-134.5).

Failures and complications

One year postoperatively, 276 patients (96.2%) had fully recovered. Eleven cases (3.8%) had re-ruptures, out of which eight were due to additional injuries during the time of recovery, like knee twisting injuries at home, the unavailability of proper care at home, and three were due to an accident. Three months later, revision surgery was performed for these cases. No significant differences were observed in the 11 patients in the re-rupture group as compared to the other patients regarding gender, age, and patient-reported outcome measures. Five cases had synovitis, which resolved without further surgical intervention. Postoperatively, seven cases had arthrofibrosis within 2-3 months, which became better once arthroscopic arthrolysis was performed. The average time to return to sports in 27 cases of professional players was one year.

## Discussion

Anterior cruciate ligament reconstruction using the all-inside technique is a well-established technique, and many studies have used different techniques to test and review its principles and outcomes [15-17]. To our knowledge, no published study has yet described in detail the all-inside ACLR technique with FiberTape InternalBrace™ or discussed it with functional outcomes and patient-reported outcomes.

The principal finding of this study was that 96.2% of patients surgically operated on using all-inside semitendinosus autograft ACL reconstruction with the FiberTape® (Arthrex) InternalBrace™ technique had excellent patient-reported outcome measures. The KOOS, WOMAC, and IKDC values all showed significant improvements, along with a significant decrease in the VAS for pain. These results observed in this study are much better than the failure rates of 25%-53% observed in studies involving primary repairs of the ACL from the 1970s and 1980s. This was one of the prime reasons why ACL reconstruction became the gold-standard surgical option for ACL tears [18,19].

ACL reconstruction can be done using various techniques involving various fixation methods; however, there is still a lot of debate about the most effective technique with a minimal anterior tibial translation that can resist rotatory loads. Internal bracing for ACL reconstruction is the latest concept that has gained popularity in recent times. Mackay et al. [10] were the first to describe the FiberTape (Arthrex) reinforcement of primary ACL repair using a synthetic suture tape. Smith et al. [20] have also described internal bracing for ACL repair in pediatric patients. Suture augmentation for all-inside adult ACLR has been reported by many authors. But not, many have recorded functional outcomes with scores like IKDC, KOOS, WOMAC, and PROMs as this study and on such many patients. Mean IKDC scores post-operatively were 96.2 (94.5-97.8) in this study, indicating patients gained knee function that was near normal with very few symptoms following ACLR, and the results are comparable with other similar studies by Parkes CW et al., who achieved a mean IKDC score of 94.4 273 (95% CI: 91.7-97.1) [11,21]. The mean Lysholm scores post-operatively at one year were 95 (93.1-96.9), suggesting that the majority of patients achieved knee function results that ranged from good to excellent post-operatively. These results are also quite similar to the study conducted by Parkes, CW et al. [11,22].

There was a significant increase in mean KOOS and WOMAC scores at the one-year follow-up. However, the limitation was that the KOOS values are recorded only at a maximum one-year follow-up, even though it has been noted in such similar studies that between 1 and 2 years, KOOS results are almost equal [23]. Additionally, ROMs were excellent at follow-ups for the vast majority of patients at one year.

The findings of decreased failure rates (3.8%) in young and active patients in our study, as compared to those in other such studies concerning ACL reconstructions, may be due to the latest technique used and proper follow-up of patient and surgeon skills [24]. Reassuringly, there was less or almost no evidence of cyst formation, erosions, or synovitis on imaging in the follow-ups or after revision surgery. This somewhat eliminates a major concern regarding complications and demonstrates the difference between traditional synthetic grafts and the internal bracing technique used in our study. Success rates (96.2%) are much better as compared to the dynamic intra-ligament stabilization ACL repair technique (80%) with five-year outcomes. Also, we have observed better results as compared to the recent ACL repair study by Gagliardi et al. done using suture tape augmentation, which showed an 81.8% success rate [25,26].

The chief objective of this study was to demonstrate that the technique is feasible without any major concerns that have been previously noted with other augmentation techniques [26]. The limitation of this current study is the lack of comparability with other ACL reconstruction procedures, as randomization was not done, and the patients that met the inclusion criteria all underwent ACL reconstruction using the FiberTape® (Arthrex) InternalBrace™ technique only.

More such clinical studies with a larger cohort and a follow-up period of at least two years are necessary. Studies with higher levels of evidence, like randomized controlled trials of ACL reconstruction using different techniques, are also needed. Such studies would be able to evaluate objectively and re-confirm the favorable results of FiberTape® (Arthrex) InternalBrace™ ACL reconstruction.

## Conclusions

It was concluded from this study that anterior cruciate ligament reconstruction with a semitendinosus autograft and the independent FiberTape® (Arthrex) InternalBrace™ technique showed better outcomes in terms of objective knee laxity testing at 12 months postoperatively and a low rate of re-rupture when compared to conventional techniques as seen in the existing literature. This technique is also seen to reduce the overall time to recovery for patients and facilitate an early return to sports. However, comparative studies are required on a larger cohort to prove it's superiority to other techniques.
